# A novel polysaccharide from the *Sarcodon aspratus* triggers apoptosis in Hela cells via induction of mitochondrial dysfunction

**DOI:** 10.29219/fnr.v62.1285

**Published:** 2018-02-15

**Authors:** Dan-Dan Wang, Qing-Xi Wu, Wen-Juan Pan, Sajid Hussain, Shomaila Mehmood, Yan Chen

**Affiliations:** 1 School of Life Sciences, Anhui University, Hefei, China; 2 Anhui Key Laboratory of Modern Biomanufacturing, Hefei, China

**Keywords:** *Sarcodon aspratus*, mushrooms, polysaccharide, apoptosis, mitochondrial dysfunction

## Abstract

**Background:**

Polysaccharides extracted from fungus that have been used widely in the food and drugs industries due to biological activities.

**Objective:**

The objective of the present study was to investigate the tumor-suppressive activity and mechanism of a novel polysaccharide (SAP) extracted from *Sarcodon aspratus*.

**Methods:**

The SAP was extracted and purified using Sepharose CL-4B gel from S. *aspratus*. The cytotoxicity of SAP on cell lines was determined by MTT method. Cellular migration assays were implemented by using transwell plates. The apoptosis and mitochondrial membrane potential (Δψm) of Hela cells were analyzed by flow cytometry. The western blot was used to determine the protein expression of Hela cells.

**Results:**

The results showed that SAP with a molecular weight of 9.01×10^5^ Da could significantly inhibit the growth of Hela cells *in vitro*. Three-dimensional cell culture (3D) and transwell assays showed that SAP restrained the multi-cellular spheroids growth and cell migration. Flow cytometry analysis revealed that SAP induced a loss of mitochondrial membrane potential (Δψm). Western blot assays indicated that SAP promoted the release of cytochrome c, increased Bax expression, down-regulated of Bcl-2 expression and activated of caspase-3 expression.

**Conclusion:**

This study suggested that SAP induced Hela cells apoptosis via mitochondrial dysfunction that are critical in events of caspase apoptotic pathways. The anti-tumor (Hela cells) activity of SAP recommended that S. *aspratus* could be used as a powerful medicinal mushroom against cancer.

In recent years, various therapeutic means have contributed to the treatment and prevention of cancers, but these methods can provoke irreversible side effects. Thus, the urgent task of the new drug research is to pursue the natural alternatives with high therapeutic properties and low toxicity. With unique advantages of nontoxic and anticancer cell activities, the polysaccharides from medicinal mushrooms have attracted global interest from pharmaceutical industries as a miraculous herbal medicine (1, 2). In particular, *Ganoderma lucidum* (3), *Lentinus edodes* (4), and *Grifola frondosa* (5) are commonly known medicinal mushrooms worldwide.

*Sarcodon aspratus* is a famous rare delicious and edible mushroom grown mainly in the Yunnan Province, China. Earlier experiments had demonstrated that the fucogalactan isolated from *S. aspratus* triggers the release of the tumor necrosis factor-α and nitric oxide from murine macrophages associated with antitumor effect (6). In addition, the polysaccharide from *S. aspratus* had immunostimulatory activity (7). Two polysaccharide fractions were separated from the mycelium of *S. aspratus* in our laboratory; they displayed strong tumor-suppressive activities with no cytotoxicity against normal cell lines *in vitro* (8). However, investigation on the *S. aspratus* has its main focus toward the characteristics and medicinal value of polysaccharides. There is little information related to biochemical mechanisms underlining the tumor cells apoptosis promotion by polysaccharides isolated from fruiting bodies of *S. aspratus.*

Mitochondria, as a vital organelle existing in almost all eukaryotic cells to supply energy for various activities, are viewed as central regulator of the decision between cellular survival and demise (9). There is a burgeoning interest in the scientific community to fully define the role of mitochondria in cellular apoptosis. There is abundant evidence to demonstrate that mitochondria can release cytochrome c and other proteins to activate caspases and trigger tumor cells apoptosis (10). The experiments presented in this article provide some guidelines for elucidating the tumor-suppressive activity and mechanism of a new polysaccharide (SAP) extracted and purified from the fruiting bodies of *S. aspratus.* Given the evidence that SAP induce Hela cells apoptosis via a mitochondrial pathway, the study will be helpful to develop novel functional foods and drugs.

## Materials and methods

### Materials and chemicals

The *S. aspratus* fruiting bodies were obtained from Yimeng Yisheng edible fungus cooperative (Yunnan, China). Human uterine cervix carcinoma cell line (Hela), human hepatoma cell line (HepG-2), human stomach cancer cell line (HGC-27), and human normal liver cell line (MRC-5) were purchased from the cell bank of Shanghai Institute of Cell Biology (Shanghai, China). Sepharose CL-4B gel was obtained from Amersham (Uppsala, Sweden). Dextran standards, standard monosaccharides, 5-fluorouracil (5-Fu), 3-(4,5-dimethylthiazol-2-yl)-2,5-diphenyl tetrazoliumbromide (MTT), 5,5′,6,6′-tetraethylimida-carbocyanine iodide (JC-1), and poly(2-hydroxyethylmethacrylate) (poly-HEMA) were obtained from Sigma (St. Louis, MO, USA). Fetal bovine serum (FBS) and RPMI-1640 medium were obtained from Wisten Biotechnology (WISTEN Co. Ltd., Nanjing); 24-well transwell plates were acquired from Corning (NY, USA). Anti-β-actin antibody, anti-caspase-9 antibody, anti-caspase-3 antibody, anti-Bcl-2 antibody, anti-Bax antibody, and anti-cytochrome c antibody were purchased from Abcam (Cambridge, UK). All the kits used in assays were supplied by Shanghai Beyotime Bioengineering Institute (Shanghai, China). All other chemical reagents used were of analytical grade.

### Preparation and purification of *S. aspratus* polysaccharide

The fruits of *S. aspratus* (100 g) were dipped into the 95% ethanol at 70°C for 2 h to remove lipid and some colored materials. After filtering, the residue was collected and immersed into distilled water (1:5, w/v) three times at 80°C for 2 h. The supernatant was concentrated to 50 mL with a rotary evaporator at 65°C under vacuum. Then, the concentrate was added to a fourfold of 95% (v/v) ethanol and kept overnight at 4°C. The precipitates were obtained by centrifugation (4,500 rpm, 10 min) and dissolved in distilled water to remove the proteins by using Sevag method (11). After that, dialyzed against distilled water, the crude polysaccharides were obtained after the freeze-drying.

The crude polysaccharides (100 mg) were loaded on Sepharose CL-4B column (2.6 × 100 cm) equilibrated with deionized water at a flow rate of 1 mL/min. The eluting fractions were monitored by high-performance liquid chromatography (HPLC) instrument (Agilent Technologies, Palo Alto, CA, USA). The relevant fraction was collected, concentrated, and lyophilized to obtain a brown polysaccharide (SAP), which was examined whether or not polysaccharide could inhibit the growth of cancer cells.

### Molecular weight determination

Molecular weight of the polysaccharide was determined by HPLC equipped with Ultrahydrogel 1000 (300 × 7.8 mm, Tosoh Corp, Tokyo, Japan) and an evaporative light scattering detector (ELSD). Standard dextrans including T-10, T-40, T-70, T-500, and T-1000 were used as molecular mass markers. Sample solution (10 mg/mL, 10 μL) was injected into each run and eluted with distilled water at 30°C with a flow rate of 1.0 mL/min.

### Infrared spectral analysis

The construction of SAP was detected by Fourier transform infrared spectrometer (FT-IR). The sample (1.0 mg) was ground with 100 mg potassium bromide (KBr) powder and then pressed into pellets for FT-IR measurement in the frequency range of 4,000–4500 cm^−1^.

### Chemical characters of the polysaccharide

The polysaccharide content of SAP was estimated by phenol-sulfuric acid method, and the protein content was detected by Coomassie Brilliant Blue G-250 method.

The monosaccharide compositions of the polysaccharide were determined using the method reported by our laboratory (12). Briefly, the SAP (10 mg) was hydrolyzed with 2 M trifluoroacetic acid (TFA, 110°C, 6 h), and the residual acid was removed with a rotary evaporator at 65°C under vacuum. The hydrolysis product monosaccharides and standard monosaccharides (including d-fructose, d-mannose, l-rhamnose, d-glucose, d-galactose, d-xylose, and l-fucose) were derivatized to be 0.5 mol/L 1-phenyl-3-methyl-5-pyrazolone (PMP) and 0.5 mol/L NaOH incubated at 70°C for 30 min. The hydrolysates were analyzed by HPLC equipped with a ZORBAX SB-C18 column (150×4.6 nm, particle size 5 m, Agilent Technologies, CA, USA). The analysis was performed using gradient elution of acetonitrile (14–20–40%) and 200 mM ammonium acetate (86–80–60%) for 0–20–30 min, respectively, with the flow rate of 1.0 mL/min and UV absorbance at 245 nm.

### Cell culture

The cell lines were cultured in RPMI-1640 medium containing 10% FBS at 37°C and 5% CO_2_ in a humidified atmosphere incubator.

### Effect of cytotoxicity of the polysaccharide

The cytotoxicity of SAP on Hela, HepG-2, HGC-27, and MRC-5 cells was determined by MTT method (13). A single cell was dispensed in a 96-well culture plate at density of 3 × 10^3^ cells per well. After 24 h, cells were incubated with various concentrations of SAP (25, 50, 100, 200, and 400 μg/mL), with 5-Fu (25 μg/mL) as positive group, whereas the control group was treated with the medium, only for 48 h. The cells were incubated with 100 μL of MTT (0.5% w/v) for 4 h at 37°C in dark. After removing the supernatant, the formazan crystals in the cells were dissolved in dimethyl sulfoxide (DMSO, 100 μL/well). Then, the plates were placed in microplate reader (Epoch-2, Biotek) and the absorbance was measured at 560 nm. The cytotoxicity of SAP was expressed as percentage of cell viability as compared to control.

### The effect of SAP restrains tumor migration ability

Cellular migration assays were implemented using 24-well transwell plates containing 8.0 μm pore membrane (14). The Hela cells (5 × 10^4^) were resuspended in serum-free medium and then added into the upper insert membrane. RPMI-1640 medium supplemented with 20% FBS was added into the bottom chamber. Cells were then treated with polysaccharide and 5-Fu for 48 h, concurrently. The cells without the treatment of SAP were used as control group. Then, the migrated cells fixed with methanol and cells localized on the bottom membrane surface. After staining with hematoxylin–eosin, the migrated cells were imaged and counted using an inverted microscope (Carl Zeiss, Oberkochen, Germany) (10× magnification, at least five fields per condition).

### Proliferation rate of multicellular spheroids of Hela cells

The cell culture flask, the bottom of which was coated with a layer of poly-HEMA thin film, was exposed to ultraviolet light for 3 h to ensure asepsis before use. The Hela monolayer cells (5 × 10^5^) were resuspended in the medium containing 5% FBS and then seeded in the cell culture flask. Cells were incubated at 37°C with 5% CO_2,_ and the culture medium was changed every other day (15). After 2 days, the Hela multicellular spheroids (MCS) could self-assemble to form three-dimensional (3D) MCS. Hela 3D tumor models maintained good structural stability for 3 days. Then, we added the water extract of SAP (200 μg/mL) and 5-Fu (25 μg/mL) to the cultures. MCS formation and growth were monitored every day; MCS of Hela cells were examined under an inverted microscope.

### Cell apoptosis analysis

To analyze apoptosis of Hela cells, an Annexin V-FITC Apoptosis Detection Kit was used (16). Hela cells were seeded at a density of 5 × 10^5^ cells per well in 6-wells plate with 1.5 mL culture medium. Twenty-four hours after cell inoculation, the SAP was added to the cultures at different concentrations (50, 100, and 200 μg/mL) and 5-Fu (25 μg/mL). After 48 h, we used Annexin V-FITC Apoptosis Detection Kit to detect the cell apoptosis. The stained cells were analyzed by FACSCalibur (BD Biosciences; Ex = 488 nm; Em = 530 nm). The data were collected and analyzed using Cell Quest pro software. At least 10,000 events were evaluated.

### Mitochondrial membrane potential

The mitochondrial membrane potential (Δψm) of Hela cells was analyzed by JC-1. JC-1 is a lipophilic fluorochrome that is used to evaluate the status of the Δψm (17). JC-1 that fluoresces in the FL-1 channel (R1), which J-aggregates, will emit red fluorescence at high membrane potential in healthy cells, and JC-1 that lacks fluorescence in the FL-2 channel (R2) is considered to correspond to mitochondrial dysfunction with a depolarized Δψm which remained green fluorescence monomers. The Hela cells were treated by the same methods as described above. They were harvested and labeled with JC-1 and then analyzed by flow cytometry. The data were collected and analyzed using Cell Quest pro software. At least 10,000 events were evaluated.

### Western blot analysis

After the treatment with SAP (0, 50, 100, 200 μg/mL) for 48 h, the Hela cells were lysed with ice-cold radioimmunoprecipitation assay (RIPA) buffer for 30 min over ice and then centrifuged at 12,000 r/min for 15 min at 4°C to obtain the cytosol fraction. In order to obtain cytochrome c protein, Cell Mitochondrial Isolation Kit was used. The protein concentration was determined with BCA Protein Assay Kit according to the manufacturer’s protocol. The proteins were denatured by boiling in 1× loading buffer, and protein samples were separated by 12% SDS-polyacrylamide gel electrophoresis (SDS-PAGE) and electroblotted onto a 0.22 μm polyvinylidene difluoride (PVDF). Subsequently, the membrane was blocked in blocking buffer (5% skim milk with 1× Tris-buffered saline [TBS] containing 0.1% Tween-20) for 3 h with shaking at room temperature, followed by incubation with the primary antibodies (cytochrome c, Bcl-2, Bax, caspase-3, procaspase-9, and β-actin) overnight at 4°C. The membrane was then washed by suspending it in washing buffer (add 5 mL of 10% Tween-20 to 1,000 mL TBS), agitated for 10 min and incubated with the appropriate secondary antibodies for 3 h at room temperature. After washing twice with TBS (15 min/time), the antibody-specific protein was visualized by enhanced chemiluminescence (ECL) detection system with ECL Kit.

### Statistical analysis

Results were expressed as mean ± standard deviation (SD). The statistical significance of difference groups was evaluated by analysis of variance, followed by Service Solutions (SPSS, version 18.0) software. A value of *p* < 0.05 was regarded to be statistically significant.

## Results and discussion

### Preparation and chemical characters of the polysaccharide

**Fig. 1:**
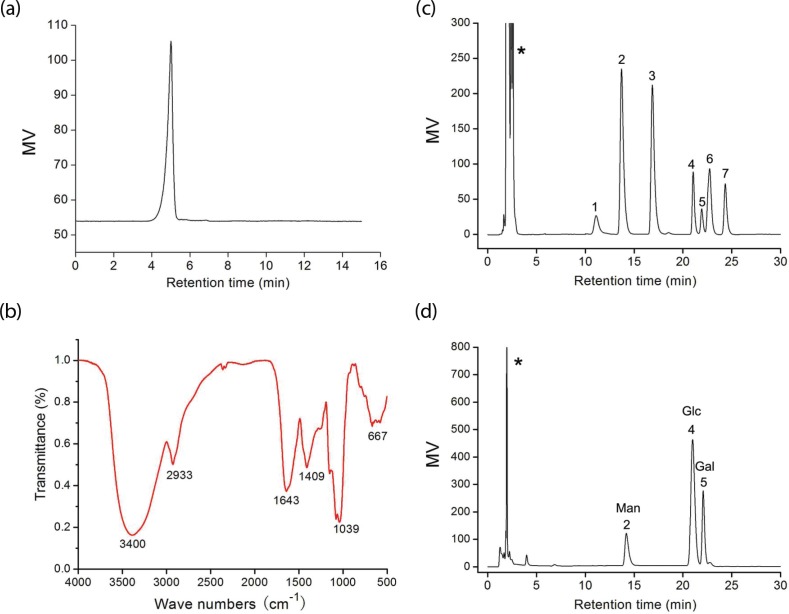
(a) HPLC chromatogram of SAP; (b) FT-IR spectrum of SAP; (c) a standard mixture of monosaccharides; (d) analysis of monosaccharide composition of SAP (*-solvent peak, 1-fructose, 2-mannose, 3-rhamnose, 4-glucose, 5-galactose, 6-xylose, and 7-fucose).

The crude polysaccharide was isolated from the fruiting bodies of *S. aspratus* and purified by Sepharose CL-4B gel. The homogeneity and average molecular weight of the SAP were estimated using an HPLC method. SAP showed single and symmetrical peaks, indicating their homogeneity (Fig. 1a). The SAP retention time was 4.63 min. The average molecular weight of SAP fractions was estimated to be about 9.01 × 10^5^ Da.

FT-IR has been shown to be a powerful tool for the identification of characteristic organic groups in the polysaccharide (Fig. 1b). The absorbance band at 3,400 cm^−1^ represented the stretching vibration of O–H in the constituent sugar residues (18). The adjacent peak at 2,933 cm^−1^ was found for the stretching vibration of C–H in the sugar ring (19). The relatively strong absorption peak at 1,643 cm^−1^ was caused by the bending vibration of C–O bonds in uronic acids. On the contrary, SAP had the absorption band centered at 1,409 cm^−1^ due to the C–H (deformation) (20). In addition, the absorbance of polysaccharides in the range 1,040–1,600 cm^−1^ was characteristic of the C–O (19). The sugar composition of SAP was identified and quantified by HPLC analysis (Fig. 1c). The results indicated that it was composed of d-galactose, d-glucose, and d-mannnose with molar ratios of 1.48:4.13:1.0. The total sugar content of SAP was about 85.68% and its protein content was 4.25%.

### Effect of cytotoxicity of the polysaccharide

**Fig. 2:**
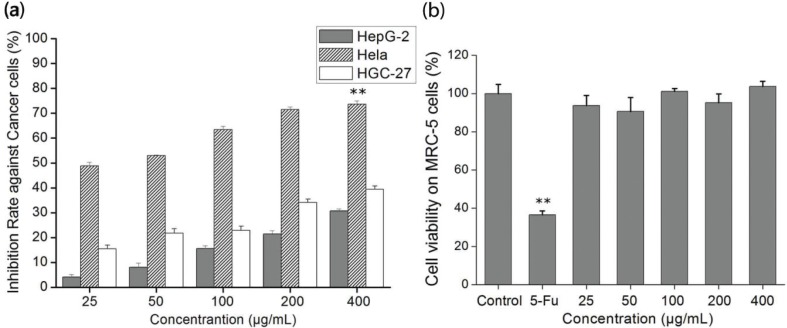
The *in vitro* growth inhibition ratio of cells by SAP. (a) Cytotoxicity of SAP in HepG-2, Hela or HGC-27 cancer cells; (b) cytotoxicity of SAP at different concentrations and 5-Fu (25 μg/mL) on MRC-5 cells. The results were expressed as means ± SD (*n* = 3) for MTT assay. (a) **p < 0.01 compared with HGC-27 cells. (b) **p < 0.01 compared with control group.

We evaluated the cytotoxicity of SAP toward Hela, HepG-2, and HGC-27 cells by MTT (Fig. 2a). The cancer cells viability obvious descent was triggered by SAP and occurred in a dose-dependent manner in comparison with the control group. The SAP presented significantly high inhibition for Hela cells that could reach 70% at the concentration of 200 μg/mL, which was in good agreement to our previous work (8). In addition, cytotoxicity of SAP toward normal cells was also determined in comparison with 5-Fu. The SAP had no cytotoxicity on MRC-5 (human normal cells) even at high concentration (400 μg/mL), but 5-Fu (25 μg/mL) killed human normal cells (Fig. 2b). Our previous study and current results indicated that polysaccharides extracted, whether from mycelium or fruiting bodies, preferably inhibit Hela cells. Based on the significant tumor-suppressive activity, Hela cells were chosen as the targeted cell line to further investigate the antitumor activity and molecular mechanism of SAP.

### SAP restrains tumor migration ability

**Fig. 3:**
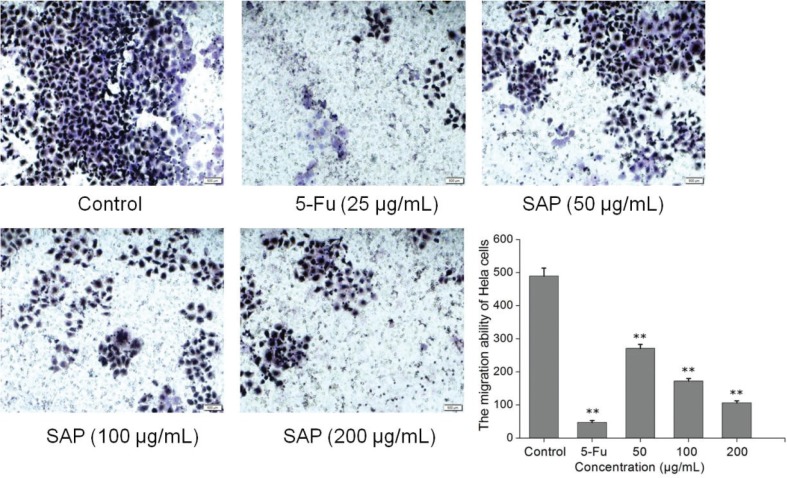
The effect of SAP restrains tumor migration ability of Hela cells. Typical photos and quantification of the transwell migration assay with Hela cells co-cultured with SAP or 5-Fu. The results were expressed as means ± SD (*n* = 3). **p < 0.01 compared with control group.

Metastasis, which is an essential character of biological behaviors in malignancies, is also the main reason for the death of tumor sufferers (21). We examined whether SAP could suppress the migration of Hela cells. The migrated cells were stained with hematoxylin–eosin; the transwell assay showed that migrated cells were significantly reduced with SAP (200 μg/mL) co-cultured for 48 h (Fig. 3). The migrated cells were counted by using an inverted microscope at six fields per condition; the data indicated that the SAP at low concentration (50 μg/mL) could suppress the cell migration compared with control (*p* < 0.05). These observations suggested that SAP significantly suppressed the migration ability of Hela cells.

### Proliferation rate of multicellular spheroids of Hela cells

**Fig. 4:**
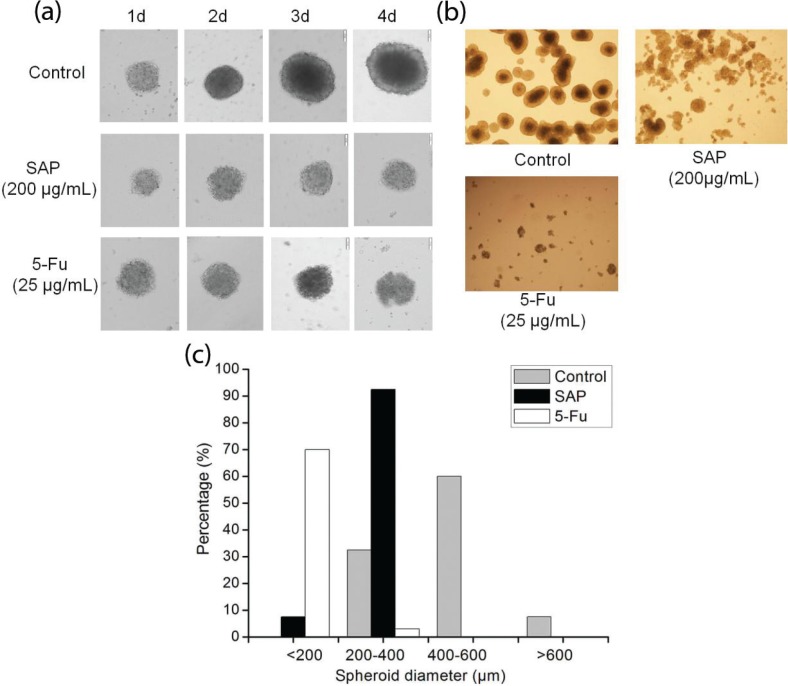
The multicellular spheroids of Hela cells were treated with 5-Fu (25 μg/mL) and SAP (200 μg/mL). (a) Cellular morphological changes during 4 days of culture in 3D constructs were observed under contrast microscope (Olympus, Japan)*.* (b) Representative photographs of the multicellular spheroids and (c) distribution of spheroid diameter in 3D Hela cells on day 6.

Three-dimensional (3D) cell culture has microenvironment characteristics of cells in living organisms, which is better than classic two-dimensional (2D) cell culture. MCS represent the most common use of cancer research (22–24). In addition, cancer cells in 3D environments exhibit stronger survival potential as well as more powerful chemoresistance to anticancer drugs (24). To validate the anticancer effect of SAP, we developed 3D tumor models based on human cancer cells. An inverted microscope was used to observe the morphology of Hela cells over a 4-day treatment with 200 μg/mL SAP and 25 μg/mL 5-Fu. Diameters of Hela MCS increased every day in control group; however, MCS in SAP-treated group were almost not increased as compared to that in the control group (Fig. 4a). To further analyze the cellular morphology in the 3D constructs, we observed a pride of MCs after 6 days (Fig. 4b). It was shown that Hela cells formed round spheroids with smooth surface and tight cell–cell connections in the control group. Image-based nano measure analysis was used to measure the spheroid’s diameter. It is obvious from Fig. 4c that MCS in the control group have shown increased diameter (>600 μm) after 6 days. On the contrary, SAP-treated MCS displayed smaller diameter (≤400 μm) as compared to the control group.

### SAP induced cell apoptosis

**Fig. 5:**
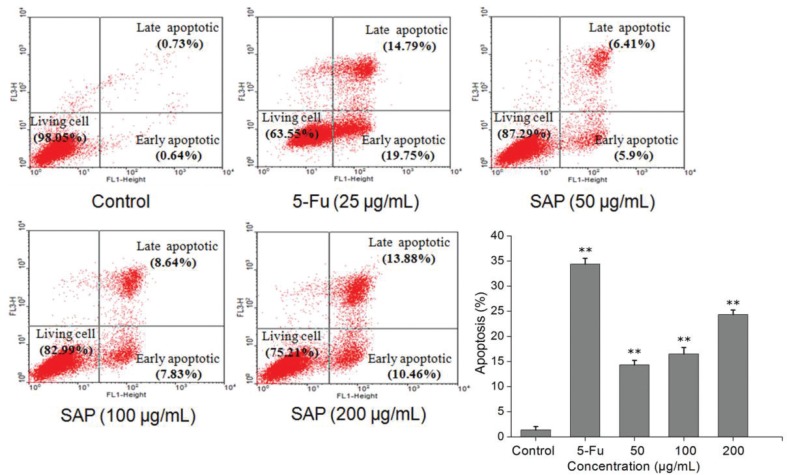
The effect of SAP induces tumor cell apoptosis of Hela cells. The cells apoptosis were monitored by flow cytometry using FACScan (BD Biosciences). The results were expressed as means ± SD (*n* = 3). **p < 0.01 compared with control group.

To investigate whether SAP induced decrease in Hela cells viability was related to cell apoptosis, Hela cells were treated with 5-Fu and different concentrations of SAP for 48 h; the cells were stained (with Annexin V-FITC and PI) and analyzed by flow cytometry. We found that low concentrations of SAP (50 μg/mL) induced cell apoptosis (12.31%) as compared to the control (1.47%) (Fig. 5). As a result of the treatment with SAP (200 μg/mL), the percentage of living cells significantly declined from 98.05% (control group) to 75.21%, which was close to the 5-Fu (25 μg/mL) effect (63.55%). In light of this, a conclusion could be drawn that SAP might induce Hela cells apoptosis.

### SAP induces the dissipation of mitochondrial membrane potential

**Fig. 6:**
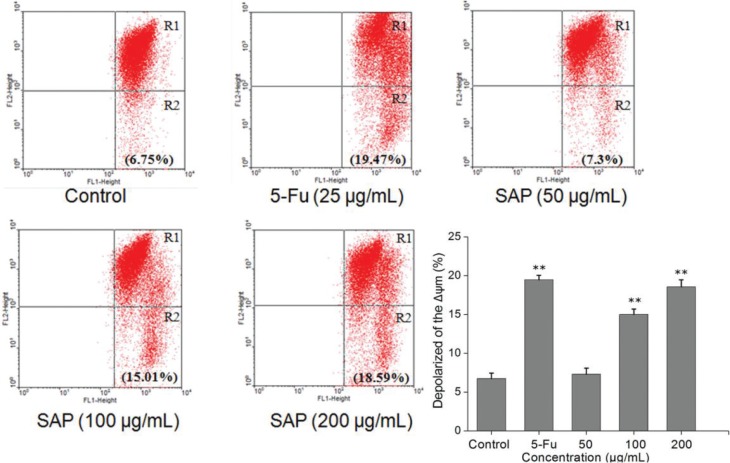
Loss of mitochondrial membrane potential of Hela cells induced by 5-Fu or different concentrations of SAP and stained by JC-1. Red/green fluorescence intensity detected by flow cytometry using FACScan (BD Biosciences). The results were expressed as means ± SD (*n* = 3). **p < 0.01 compared with control group.

Recent studies have shown that mitochondrial metabolism is a research field that has development for cancer therapy (25). Apoptosis is frequently associated with depolarization of the mitochondrial membrane potential (Δψm). The majority of cells in healthy cultures will have a polarized Δψm; loss of Δψm is an early event in the apoptotic process (26). In this study, the cells were stained with JC-1 according to the protocol and analyzed by flow cytometry. The change of Δψm was observed in treated Hela cells (Fig. 6). Compared to control, the proportion of the green fluorescence of cells increased from 6.75 to 18.59% after treatment with 200 μg/mL of SAP. Taken together, the results indicated that SAP could induce the dissipation of mitochondrial membrane potential (Δψm) which facilitated the apoptosis of Hela cells.

### SAP induced cells apoptosis via regulation of cytochrome c and Bcl-2 family

**Fig. 7:**
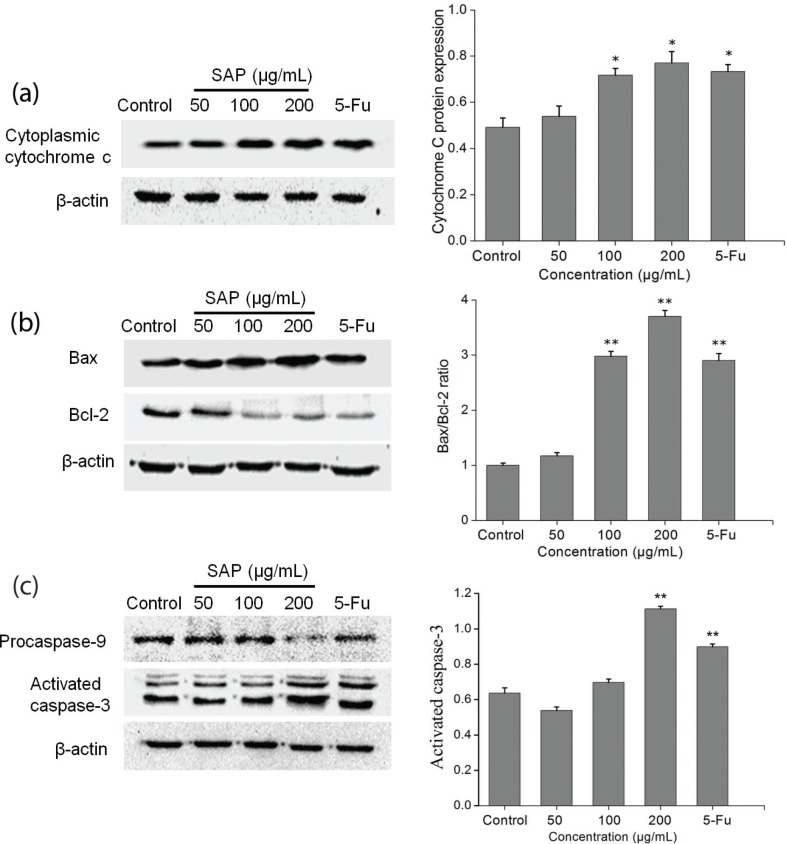
Cytochrome c, Bcl-2, Bax, procaspase-9, and caspase-3 expression were analyzed by western blot. (a) SAP regulated the Hela cells release of cytochrome c from mitochondria and (b) Bcl-2 protein family. (c) SAP regulated the Hela cells procaspase-9 and activated caspase-3. The results were expressed as means ± SD (*n* = 3). *p < 0.05 and **p < 0.01 compared with control group.

A reduction in the mitochondrial membrane potential is usually accompanied by release of cytochrome c into the cytosol. The release of cytochrome c from mitochondria is a particularly important event in the induction of apoptosis (27). Therefore, we examined the amount of cytochrome c in the cytoplasm using western blotting technique. After 48 h, a significant (p<0.05) increase in cytosolic cytochrome c was observed (Fig. 7a).

On the one hand, this mitochondrial-mediated apoptosis pathway is regulated by the pro-apoptotic effectors (Bax, Bak, Bid, Bad, etc.) and the anti-apoptotic effectors (Bcl-2, Bcl-k, Bcl-w, etc.). The Bax serves as a major regulator to accelerate the permeability of mitochondria and release of cytochrome c, whereas the Bcl-2 resides in the mitochondrial wall and protects the integrity of mitochondria against cell apoptosis (28). On the other hand, Bcl-2 expression does not influence Bax expression but inhibit activated caspase-3 expression in a dose-dependent manner (29). Therefore, the ratio of Bax/Bcl-2 is one of the decisive factors that determine cell apoptosis. With the increase in SAP concentration, the expression level of Bcl-2 gradually decreased, whereas the expression level of Bax gradually increased (Fig. 7b). The results suggested that SAP could promote apoptosis by elevating cytochrome c and Bcl-2 family.

### Caspase 3 contributes to the SAP-induced Hela cells apoptosis

Once cytochrome c is released from the mitochondria into the cytoplasm, it binds to Apaf-1 and subsequently recruits procaspase-9 to the apoptosome which can activate caspase-9 (30). The caspase-9 cleaves and activates downstream effector caspase-3. Caspase-3 is regarded as a crucial mediator of programmed cell apoptosis which has a prominent execution in the apoptotic signaling pathways (31). To investigate SAP-induced apoptosis in Hela cells, western blot technique was used to detect the expression level of activated caspase-3 and procaspase-9. The results (Fig. 7c) indicated that during treatment with 200 μg/mL SAP, the activation of caspase-3 prominently increased (reaching 1.77 times compared with control group), while procaspase-9 decreased dramatically. The above-mentioned results presented that SAP could regulate the activities of caspase-9 and caspase-3 and induce the Hela apoptosis subsequently.

## Conclusions

**Fig. 8:**
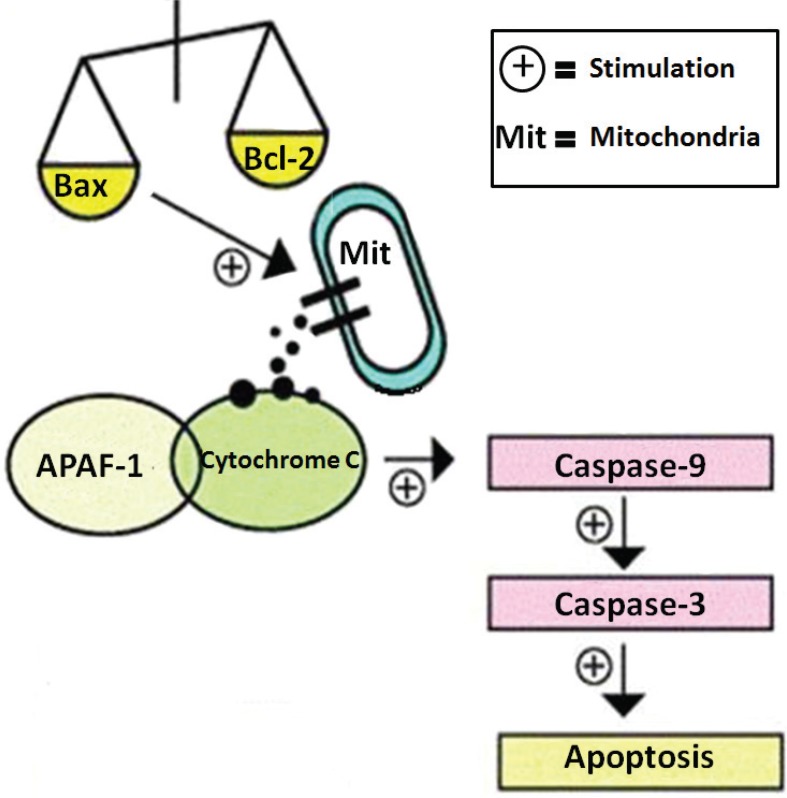
Possible apoptotic mechanisms of SAP in Hela cells.

In this study, we isolated, purified, and characterized the novel polysaccharide from the fruiting bodies of *S. aspratus*. We evaluated the cancer cells cytotoxicity of SAP on tumor cells, *in vitro*. The results showed that, no matter at three-dimensional (3D) cell culture or two-dimensional (2D) cell culture, SAP (50 μg/mL) could significantly restrain the growth of tumor cells with no cytotoxicity against normal cells. Our study investigated the apoptotic pathways by SAP in Hela cells. Experimental results suggested that SAP caused mitochondrial dysfunction by increased expression of Bax and decreased expression of Bcl-2 which prompted release of cytochrome c from the mitochondria to the cytosol. These pro-apoptotic effectors caused recruitment of procaspase-9 which can activate caspase-9. The caspase-9 cleaves and activates downstream effector caspase-3 resulting in programmed cell apoptosis. These results speculate that SAP induces tumor cells apoptosis at least in part through the activation of caspase-3 and mitochondrial pathway (Fig. 8). In a nutshell, *S. aspratus* could be used as a potential medicinal agent for the treatment of cervical carcinoma.
